# A Case of Spinal Irritation, Attended with Loss of Vision; Recovery

**Published:** 1852-08

**Authors:** Charles Fishback

**Affiliations:** Shelbyville, Ind.


### WESTERN JOURNAL

OF

### MEDICINE AND SURGERY.

---

AUGUST, 1852.

---

Art. I.—*A Case of Spinal Irritation, attended with loss of Vision; Recovery.* By Charles Fishback, M. D., of Shelbyville, Ind.

John Smith, æt. 30, temperament sanguineo-nervous, the latter predominating decidedly; florid complexion, light hair, blue eyes, brain large anteriorly and superiorly; health feeble in childhood, but generally good since puberty. At about five years of age was blind for several weeks, cause unknown, eyes were red, but not painful. The physician who was called in, said he could save the right eye, but not the left, by putting in it a powder. This caused great swelling, threatening the bursting of the globe, and violent pain. The sight of the left eye was restored, but the right was of no use until the winter of 1850 and '51, after the loss of vision of the left eye, when the function of the right eye gradually and steadily improved, until in July, 1851, the pain which had been previously confined to the left side invaded the right, and then impairment of vision of right eye occurred, and progressed to complete loss early in November, 1851. He has a sister older than himself who was blind for some time during her youth, but has not been since.

Mr. S. is a farmer by occupation; has been married four years, and has two children. In September, 1850, was attacked with pain of the left side of his head and face, extending exactly to the mesial line anteriorly and posteriorly, and embracing all of that side of the head and face excepting the globe of the eye. About the middle of November, 1850, having taken severe cold from getting wet, violent pain appeared in the globe of the left eye, and in a few days, vision of that eye became impaired, the globe assuming a more prominent appearance, and becoming, to his friends, larger than formerly but without redness; vision entirely lost in about two months. About the time of taking cold in November, Dr. McGeaughy was called in. Prescribed an active cathartic, to be followed by quinia and Ferri carb. in large doses, without sensible diminution of pain, and causing great irritability of stomach, attended with distressing flatulency and rejection of every article of food and drink. The quinia and iron were continued at intervals until May. Large portions of opium and morphia were given during this period, to allay the almost insupportable pain, inducing the ordinary unpleasant effects of opium in large and successive doses, in an aggravated degree. Dr. M. also gave guiac for rheumatism, which he said was a complication. There was pain of the shoulders and upper third or more of spinal column.

In January, 1851, there was a remission of the pain for a short time. In April, there was entire absence of pain for two or three weeks, and he went to work. During the latter part of winter and early in the spring, resort was had to electro-magnetism, sometimes with relief to pain, but not at others.

Towards the close of May, 1851, he visited Cincinnati, and consulted Prof. Mussey, whose written opinion and prescription are as follows, viz: "There is some reason to hope for relief from a suitable diet, with some medicine. Never let the bowels become costive. If there be a tendency that way, take at bed-time of rhubarb and charcoal, each 10 grs., and ginger 5 grs., mixed together; or take occasionally at bed-time, one of the following pills, viz: Ext. colocynth comp. ℈iiss, sapo. castil. ℈ss, oil Croton, gtt., j. m. ft. pills, 12. One hour after eating, take, in a tablespoonful of rain water, four drops of the solut. Fowleri mineralis; continue it for five or six weeks, unless the vision is very much improved in that time, or some of the specific effects of the article should manifest themselves. In either case, its use may be suspended. Use no tobacco in any form, nor strong drink, nor coffee. Avoid the eating of meat. Perhaps you may take a boiled egg once a day, if it be found to agree with the stomach. Drink weak black tea, or water. Eat no bread that is less than one day old. Never eat anything between meals."

There had been a tendency to constipation all along. No sensible effect was produced by the solution Fowleri mineralis, though taken according to directions. Only one or two of the pills was ever taken. There was comparative comfort from middle of May to middle of July, at which time pain attacked the right side of his head and the right eye. He now came under the care of Dr Wolf, who removed six fangs, and ordered solution sulphate of magnesia to keep the bowels open, and pills containing a mercurial, which soon produced slight ptyalism, without any improvement, but on the contrary great prostration, so as to require aid in changing his place from bed to chair. He also gave, night and morning, a pill containing strychnine. He also ordered a resumption of the quinia and iron. No benefit followed, but general and complete prostration.

He came under my notice October, 1851, very weak, much reduced in flesh, pulse feeble, and irritable, and a little over 100 beats a minute. Tongue furred and deeply fissured in every direction over its entire surface. Bowels torpid, gastric flatulency great, and frequently joined with acidity. Appetite poor and irregular. No enlargement of abdominal viscera perceptible, nor tenderness, except of epigastrium, which is exceedingly tender; urine high colored, and variable in quantity. Excessive tenderness over the entire spinal column, a little greater in the upper two-thirds of the dorsal region than elsewhere; complains much of pain and weakness of the back. Pain of the right side of the head constant and severe, but variable in degree, being greatest when flatulency and acidity of the stomach prevailed. Has been troubled with almost icy coldness of feet since about the time that stomach became irritable, or nearly a year, and is most felt when pain of head is severest. For sometime past, cannot sleep more than two or three hours in twenty-four, and often less; sometimes none at all. Sense of fatigue throughout the muscular system, but no pain or tenderness. Pupils dilated almost to their full extent, but contracting considerably in a strong light. Cornea clear; conjunctival color not perceptibly altered. A very few tortuous vessels over the sclerotic portion of the globe. Neither the circumcorneal zone, nor the straight, and fine vessels indicative of sclerotitis visible with a good pocket magnifier. Globes prominent, with fullness of palpebræ, upper and lower; vision of the left eye entirely gone. Can see a little wlth right eye. Coldness of extremities, superior and inferior, general, intense, and almost constant.

Regarding the state of vision as dependent mainly upon disease of the optic nerve, or of that part of the brain from which it arises, and *this partially*, upon the state of the constitution, I directed treatment to the latter, First:

Kept the bowels in a soluble state, with pill colocynth, compos., of the United States Dispensatory, modified by the addition of gamboge and rhubarb. Quieted his nervous system and stomach, by scarified cups over spine and stomach, followed by emplast pix. Burgund.; hot pediluvia with mustard; nitro-muriatic acid to entire cutaneous surface, and iij grain pills of ext. hyosciamus, repeated every hour or two, until the desired effect is obtained. Endeavored to correct acidity of stomach with Bismuthis subnitrat., which was only partially successful, neutralizing, but not preventing acidity. During the month of October, his improvement was steady, and much more rapid than I had dared to hope for. Though not perfectly free from pain of the head, he was comparatively so. Sleeps sufficiently, and it is refreshing, and much of the time without the anodyne. Pulse became slower, about 80 per minute, stronger and calmer. Bowels soluble, and daily moved, without medicine after the first week. Tenderness of spine and epigastrium scarcely perceptible. Strength much increased; spirits revived and cheerful. Vision of right eye much improved, without sensible change in the appearance of the eye.

October 24th. Introduced a seton in the nape, and kept it running for about six weeks. A week or ten days after its introduction, he contracted a severe cold, from exposure during the greater part of a night, caring for a sick horse. An increase of the general malaise, and of the head in particular, followed. Ung. veratria to forehead, mastoids and nape; Ung. tart. antimony to epigastric and lumbar spinal regions, after scarified cups and pills argent. oxidi, grs. ij, ter die, November 12th, and subsequently, with benefit.

November 18th. Having suddenly risen from his seat, and stepped to the door, he fell to the floor, and was insensible and apparently breathless for some minutes; face pale. About an hour afterwards, he commenced talking, as if he had been asleep, without recollection of anything after reaching the door. This I suppose to have been syncope. For a week afterwards the pain, coldness of extremities, and restlessness, were greatly increased, with loss of appetite. There were also occasional flushings of the face, with a sense of fullness about the head. With the renewal of pain, came diminution of vision, until in a few days, it was entirely lost.

He was at home nine miles from town on the 18th, and until the 28th. I visited him on the 22d, and found him suffering beyond expression Ordered, ℞. syrup sarzae comp. (U. S. Dispensatory,) ℨj, potassii iodidi, grs. v, ter die. Caused slight nausea, but was continued. Pills ext. hyosciamus, and frictions to cutaneous surface, with hot pediluvia. He got better and came to town on the 28th. The next day, I applied one leech to the scalp near the anterior and inferior portion of the right parietal bone, and another over the occipital bone, about an inch above and behind the mastoid, of same side; at both of which points there was considerable tenderness. Ordered, ℞. magnesia sulphatis, ℥i, ant. tart. grs. i, Vin. colchici f. ℥ss; aquae ℥iv, M. S. a table spoonful sufficiently often to insure two or three dejections daily.

December 3d. Two leeches to anterior and one to posterior points of scalp as before.

December 5th. Improved feeling to-day, for the first time since about the 20th of November, distinguished day-light.

December 8th. Still further improved in feeling, appetite and strength—quite free from pain. Sleeps well without anodyne; bowels regularly open without medicine, some flatulence yet, but slight; can see persons moving about the room.

Feb'y 20th. He has steadily improved; can see to ride over the country, and to walk about town; as heavy as ever he was, and feels as well as he ever did.

Shelbyville, Ind., July, 1852.

